# Baicalein alleviates long-term cognitive impairments induced by repeated neonatal sevoflurane exposure via inhibition of cortical microglial TLR4/NF-kB signaling

**DOI:** 10.1016/j.bbih.2025.101126

**Published:** 2025-11-03

**Authors:** Yingqiao Niu, Qiuting Zeng, Yan Yang, Wenbo Liu, Hui Zhang, Shuyu Chen, Xiaomin Li

**Affiliations:** a Department of Anesthesiology, Nanjing Drum Tower Hospital, The Affiliated Hospital of Nanjing University Medical School, Nanjing, China; b Department of Anesthesiology, Zhongda Hospital, School of Medicine, Southeast University, Nanjing, China; c Department of Anesthesiology, Zibo Central Hospital, Zibo, China

**Keywords:** Baicalein, Sevoflurane, Microglia, TLR4/NF-κB signaling, Neuroinflammation, Cognitive impairment

## Abstract

Sevoflurane, a widely used pediatric anesthetic, has been associated with long-term cognitive impairments following repeated neonatal exposure. Baicalein, a flavonoid derived from Scutellaria baicalensis, exhibits neuroprotective and anti-inflammatory properties. This study evaluated whether baicalein can mitigate sevoflurane-induced neuroinflammation and cognitive deficits in developing rats. Neonatal rats were exposed to sevoflurane (3 %, 2 h/day) from postnatal day (P) 6 to P8. In the intervention group, baicalein (50 mg/kg) was administered intraperitoneally from P6 to P8 and orally via drinking water from P21 to P35. Cognitive performance was assessed between P35 and P40 using the open field test, novel object recognition, and fear conditioning paradigms. Brain tissues were collected for Western blot, ELISA, and immunofluorescence analyses. Baicalein treatment significantly attenuated sevoflurane-induced deficits in memory and learning, particularly in the novel object recognition and fear conditioning tasks. Mechanistically, baicalein inhibited microglial activation, reduced cortical expression of TLR4 and phosphorylated NF-κB p65, and decreased pro-inflammatory mediators including iNOS, IL-1β, and IL-6 at P8. These findings indicate that repeated neonatal sevoflurane exposure impairs cognitive function via microglial-mediated neuroinflammation, and that baicalein's neuroprotective effect is at least partly attributable to modulation of cortical microglial activity via TLR4/NF-κB signaling. This study highlights baicalein as a promising therapeutic strategy to prevent long-term neurodevelopmental deficits in neonates.

## Highlights

• Baicalein suppresses microglia-mediated inflammation via the TLR4/NF-κB pathway in neonatal rat cortex.
• Repeated neonatal sevoflurane exposures lead to long-term cognitive impairments.
• Baicalein significantly alleviates sevoflurane-induced neuroinflammation and cognitive deficits.
• Targeting neuroinflammation may offer a novel strategy to mitigate early-life anesthesia-related brain injury.

## 1 Introduction

The increasing use of general anesthesia in infants has raised concerns regarding its potential risks to neurodevelopment. Sevoflurane, the most commonly used inhalational anesthetic in pediatric and neonatal anesthesia due to its rapid onset, low blood-gas partition coefficient, and minimal airway irritation (Goyagi, 2018). Preclinical studies indicate that neonatal sevoflurane exposure induces neuronal apoptosis (Yang et al., 2025; Jeong-Rim Lee et al., 2021), alters synaptic plasticity (Jiang et al., 2023), and results in long-term cognitive impairments (Warner et al., 2018). Clinically, repeated anesthesia and surgery before age three do not impair general intelligence but are associated with deficits in fine motor skills and processing speed in late childhood (Feldman, 2022). These findings highlight the need for strategies to mitigate anesthesia-induced neurodevelopmental risks.

Neuroinflammation, particularly microglial activation, is increasingly recognized as a key mechanism underlying sevoflurane-induced neurotoxicity. Prolonged neonatal exposure alters microglial morphology, including increased branching, total branch length, arborization area, and structural complexity, observed 14 days post-exposure (Cheng et al., 2025). Microglial activation is a crucial factor in the development of several neurodegenerative disorders, such as Parkinson's disease (PD) (Sen et al., 2022), multiple sclerosis (MS) (Zhu et al., 2022), Alzheimer's disease (AD) (Tan et al., 2023), postoperative cognitive dysfunction (POCD) (Sellgren et al., 2019), and schizophrenia (Zuo et al., 2022). In rats, sevoflurane-induced cognitive impairments are associated with impaired neural stem cell (NSC) proliferation and neurogenesis mediated by microglial activation, neuroinflammatory, and suppression of the VEGF receptor 2 (VEGFR2) signaling pathway (Sun et al., 2016). Despite these insights, the molecular mechanisms by which sevoflurane drives microglial activation remain incompletely elucidated.

Beyond neuroinflammation, neonatal sevoflurane exposure disrupts redox homeostasis and neuronal signaling. Reported alterations include increased superoxide production and NADPH oxidase subunit p22ˆphox, reduced glutamatergic neurons in the basolateral amygdala, and enhanced depolarizing activity of γ-aminobutyric acid type A receptors (Satomoto et al., 2018; Zhang et al., 2016; Chen et al., 2022; Poltorak et al., 1998). Our previous work further implicates endoplasmic reticulum stress-induced autophagy in neurons and microglia as a contributor to postoperative cognitive dysfunction in neonatal rats (Zhang et al., 2023). However, whether microglial activation directly mediates long-term cognitive impairments after repeated sevoflurane exposure remains unresolved.

Among microglial signaling pathways, Toll-like receptor 4 (TLR4) plays a pivotal role in innate immune responses by activating NF-κB and promoting pro-inflammatory cytokine production (Park and Lee, 2013). Pharmacological or genetic inhibition of TLR4/NF-κB signaling can reduce microglial activation and neuronal apoptosis (Tan et al., 2018), but its contribution to sevoflurane-induced cognitive deficits has not been determined.

Baicalein (5,6,7-trihydroxyflavone), a flavonoid derived from Scutellaria baicalensis, exhibits potent anti-inflammatory and neuroprotective properties (Jadhav and Kulkarni, 2023; Won et al., 2021; Ali et al., 2020). It mitigates systemic and neuroinflammation by modulating the TLR4/NF-κB pathway in models of sepsis and vascular dementia (Yilmaz et al., 2023; Song et al., 2024). Whether baicalein can prevent long-term cognitive impairments induced by repeated neonatal sevoflurane exposure, and whether this effect involves TLR4/NF-κB modulation, remains unknown. In this study, we investigated the potential of baicalein to alleviate sevoflurane-induced neuroinflammation and cognitive dysfunction in neonatal rats via TLR4/NF-κB signaling.

## 2 Methods and materials

### 2.1 Animals

A total of 168 male Sprague Dawley rats (postnatal day 6, P6; 10–13 g), born to 12-week-old pregnant dams, were used for all experiments. On P6, pups from each litter were randomly allocated into four groups (n = 42 per group): control group (CON): rats were exposed to 30 % oxygen for 2 h daily for three consecutive days; baicalein group (CON + BA): rats received intraperitoneal injections of baicalein (≥98 % purity, reagent grade, 50 mg/kg, Ronghe Co., Shanghai, China) for three consecutive days, followed by oral administration starting at P21 after cage separation. sevoflurane group (SEV): rats were exposed to 3 % sevoflurane in 30 % oxygen for 2 h daily for three consecutive days; sevoflurane + baicalein group (SEV + BA): rats were exposed to 3 % sevoflurane in 30 % oxygen as above and treated with baicalein via intraperitoneal injections during exposure and oral administration starting at P21. Animals were allocated for behavioral testing (n = 48), Western blot analysis (n = 40), ELISA analysis (n = 40) and immunohistochemical analysis (n = 40). All experimental procedures were approved by the Laboratory Animal Care and Use Committee of Southeast University (Ethical permission code: 20210301071). Rats were housed under controlled environmental conditions with free access to food and water, maintained on a 12 h light-dark cycle at a constant temperature of 24–25 °C.

### 2.2 Anesthesia

**Fig. 1:**
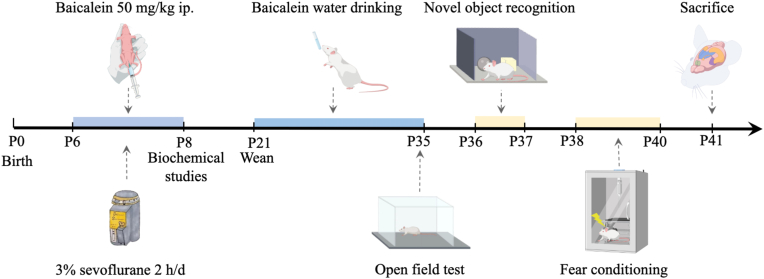
The experimental timeline. Schematic representation of the experimental timeline. OF: open field; NOR: novel object recognition; FC: fear conditioning. Neonatal rats were exposed to 3 % sevoflurane for 2 h daily from postnatal day 6 (P6) to P8 and concurrently received intraperitoneal injections of baicalein (10 mg/kg/day). From P21 to P35, rats were given baicalein-supplemented drinking water.

From P6 to P8, neonatal rats were anesthetized in a transparent, semi-enclosed respiratory loop chamber with desiccant and carbon dioxide adsorbent. Anesthesia was induced and maintained with 3 % sevoflurane in 30 % oxygen (2 L/min, National Drug Administration: H20070172, Lot No. 14071631, Shanghai Hengrui Pharmaceutical Co., China). Following recovery of the righting reflex (Qiu et al., 2023), pups were returned to their home cages and remained with their dams until weaning at postnatal day 21 (P21). To ensure airway patency and minimize the risk of posterior tongue displacement, pups were placed in the right lateral position and allowed to breathe spontaneously during anesthesia. The gas concentration within the chamber was continuously monitored using an anethetic gas monitor (GE Datex-ohmeda, Tewksbury, MA, USA). Control animals were placed in the same chamber system and exposed to 30 % oxygen at the same flow rate (2 L/min) without sevoflurane. The experimental timeline is illustrated in Fig. 1.

### 2.3 Drug administration

Baicalein (BA, ≥98 % purity) was obtained from Shanghai Ronghe Co. (Shanghai, China). For intraperitoneal administration, BA was dissolved in dimethyl sulfoxide (DMSO) and stored at −80 °C until use. Neonatal rats received daily intraperitoneal injections of BA (50 mg/kg) from P6 to P8 (Yilmaz et al., 2023). For oral administration, BA was dissolved in ethanol and supplied in the drinking water beginning on P21 and continuing until P35. The final ethanol concentration in the drinking solution was adjusted to 0.06 %. Based on an average daily water intake of approximately 5 mL per rat, the oral BA dosage was estimated to be 0.5 mg per rat per day.

### 2.4 Open field test (OFT)

At P35, the open field test was performed in a vertical open box (100 × 100 × 40 cm). Each rat was placed in the center facing the wall, and its free movement was recorded for 5 min. The total distance traveled and the time spent in the central compartment were analyzed using an automated video tracking system (XR-XZ301, Shanghai Xinruan Information Technology Co., Ltd., Shanghai, China). The apparatus was cleaned with 75 % ethanol before and after each test to eliminate residual odors.

### 2.5 Novel object recognition (NOR)

The NOR test was conducted as previously described (Owen et al., 2022) at P36-37, following the OFT. The procedure consists of three phases: habitation training, and testing. In the habituation phase (Day 1), rats were placed in a chamber (50 × 50 × 40 cm) and allowed to freely explore the environment for 5 min without objects. During the training phase (1 h later), two identical objects (shape, size and color) were placed in the opposite corners of the chamber, 10 cm from the side walls. Rats were then placed in the chamber and allowed to explore for 5 min. In the testing phase (Day 2), one of the familiar objects was replaced with a novel object that different in shape, color, and texture. The testing phase lasted 5 min for each animal. Object interaction was defined as nose orientation within 2 cm of an object or direct physical contact. The discrimination index (DI) was calculated as: (time interacting with the novel object-time interacting with the familiar object)/(time interacting with the novel object + time interacting with the familiar object) × 100.

### 2.6 Fear conditioning test (FCT)

At P38–P40, a fear conditioning paradigm was conducted to assess associative learning and memory. The protocol consisted of training, contextual testing, and cued testing. During the training phase, rats were placed individually into a conditioning chamber (30 × 30 × 45 cm) and allowed to explore freely for 3 min. An auditory cue (30 s, 70 dB, 3000 Hz) was presented, immediately followed by a foot shock (2 s, 0.5 mA). After 30 s, the rats were returned to their home cages. Twenty-four hours later, a contextual memory test was performed. Rats were re-exposed to the same chamber for 3 min without any stimuli. On the following day, a cued memory test was conducted. The chamber was modified with floor dividers and colored panels to create a novel context. Rats were placed in this novel environment for 180 s without any stimuli, followed by 180 s of auditory cue presentation (70 dB, 3000 Hz).

Freezing behavior, defined as the absence of all movement except respiration, was recorded as an index of fear memory. Data were collected and analyzed using an automated video tracking system (XRXC404; Shanghai Xinruan Information Technology Co., Ltd., Shanghai, China). The chamber was thoroughly cleaned with 75 % ethanol between trials to prevent olfactory interference.

### 2.7 Immunofluorescence staining

Rats (n = 5 per group, P8 and P41) were anesthetized with sodium pentobarbital (50 mg/kg, i.p.) and perfused transcardially with ice-cold PBS followed by 4 % paraformaldehyde (PFA). Brains were post-fixed in 4 % PFA overnight and then cryoprotected in 30 % sucrose in PBS for 3 days until they sank. Cryoprotected brains were embedded in Optimal Cutting Temperature (OCT) compound, rapidly frozen, and coronally sectioned at 25 μm using a cryostat (Leica CM3050S).

For immunofluorescence, sections were washed with PBS, permeabilized with 0.3 % Triton X-100 in PBS, and blocked with 10 % goat serum for 1 h at room temperature. Sections were then incubated overnight at 4 °C with the following primary antibodies: rabbit anti-Iba-1 (1:200, 10904-1-AP, Proteintech), mouse anti-TLR4 (1:50, sc-293072, Santa Cruz), and mouse anti-p-NF-κB p65 (1:50, sc-136548, Santa Cruz). After washing, sections were incubated with Alexa Fluor 488 or 594 conjugated secondary antibodies (Thermo Fisher Scientific) for 2 h at room temperature. Nuclei were counterstained with DAPI antifade medium (Beyotime), and images were acquired using a fluorescence microscope (MF31, Mshot).

### 2.8 Western blotting

Rats (n = 5 per group, P8 and P41) were anesthetized with sodium pentobarbital (50 mg/kg, i.p.) and decapitated. The prefrontal cortex was collected and stored at −80 °C. Tissues were lysed in RIPA buffer containing PMSF (100:1), incubated on ice for 20 min, and centrifuged at 12,000 rpm for 15 min at 4 °C. Protein concentrations were determined using a BCA assay (NCM). Equal amounts of protein were denatured, separated by SDS–PAGE, and transferred to PVDF membranes (Millipore). After blocking, membranes were incubated overnight at 4 °C with primary antibodies against CD68 (1:1000, Proteintech), GFAP (1:500, Santa Cruz), iNOS (1:2000, Proteintech), TLR4 (1:500, Santa Cruz), p-NF-κB p65 (1:500, Santa Cruz), and GAPDH (1:500, Santa Cruz). Following incubation with HRP-conjugated secondary antibodies, signals were visualized using ECL reagent and quantified with ImageJ.

### 2.9 ELISA

Rats (n = 5 per group, P8 and P41) were anesthetized with sodium pentobarbital and sacrificed by cervical dislocation. The prefrontal cortex was collected and stored at −80 °C. Enzyme-linked immunosorbent assay (ELISA) was performed to measure cortical IL-1β and IL-6 levels using commercial kits (IL-1β: PI303, Beyotime; IL-6: PI328, Beyotime) following the manufacturer's instructions (Arioz et al., 2019). Optical density was measured at 450 nm using a microplate reader (Varioskan Flash, Thermo Fisher Scientific, USA).

### 2.10 Statistical analysis

All data are presented as mean ± standard deviation (SD). Statistical analyses were performed using GraphPad Prism 9.0 (GraphPad Software, San Diego, CA, USA). Behavioral data involving multiple groups (e.g., CON, SEV, SEV + BA) were analyzed by two-way ANOVA to assess the effects of treatment and drug, followed by Tukey's post hoc test for multiple comparisons. Biochemical assays, including Western blot and ELISA, and immunofluorescence quantification (e.g., Iba-1, TLR4, p-NF-κB) were analyzed similarly using two-way ANOVA with Tukey's post hoc test. For immunofluorescence, cell counts or fluorescence intensities were averaged across at least three sections per animal. A *P*-value <0.05 was considered statistically significant.

## 3 Results

### 3.1 Baicalein alleviates cortical microglial activation induced by sevoflurane exposure in postnatal day 8 rats but not in postnatal day 41 rats

**Fig. 2:**
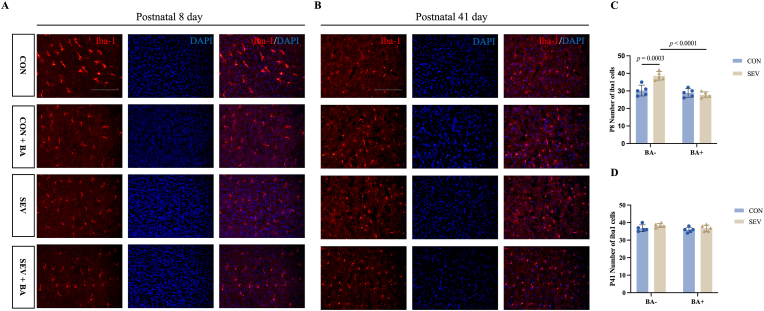
Baicalein alleviates cortical microglial activation induced by sevoflurane exposure in postnatal day 8 rats but not in postnatal day 41 rats. A: Representative immunofluorescence images showing microglial activation in the cortex of P8 rats, labeled with Iba-1. Scale bar = 100 μm. B: Representative immunofluorescence images showing microglial activation in the cortex of P41 rats, labeled with Iba-1. Scale bar = 100 μm. C, D: Quantitative analysis of Iba-1-positive microglia in the cortex of P8 and P41 rats. Data are presented as means ± standard deviation (S.D.), with individual data points representing separate animals (n = 5 per group).

To assess the impact of repeated sevoflurane exposure on cortical microglial activation of neonatal rats, immunofluorescence staining with Iba-1 was performed. In P8 rats, repeated sevoflurane exposure significantly increased the number of Iba-1-positive microglia compared with controls (Fig. 2A–C: CON [30.20 ± 3.11] vs. SEV [38.60 ± 2.41], *P* = 0.0003). Baicalein treatment significantly reduced the number of activated microglia (Fig. 2A–C: SEV [38.60 ± 2.41] vs. SEV + BA [27.80 ± 1.64], *P* < 0.0001). In contrast, P41 rats showed no significant group differences (Fig. 2B–D: CON [36.80 ± 1.92] vs. SEV [38.40 ± 1.14], *P* = 0.4227; SEV [38.40 ± 1.14] vs. SEV + BA [36.80 ± 1.79], *P* = 0.4227).

### 3.2 Baicalein inhibits sevoflurane induced cortical inflammatory responses in neonatal rats but not adolescent rats

**Fig. 3:**
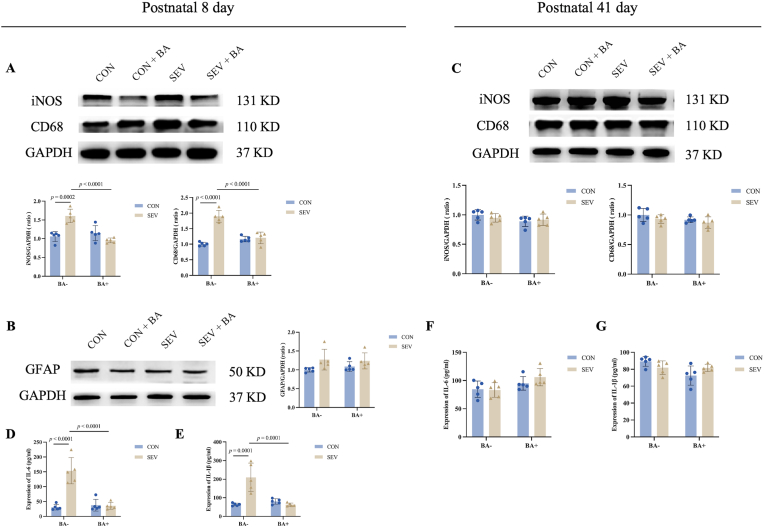
Baicalein inhibits sevoflurane induced cortical inflammatory responses in neonatal rats but not adolescent rats. A: Western blot analysis of iNOS and CD68 in the cortex of postnatal day 8 (P8) rats. Top: representative blot images; Bottom: quantitative analysis of protein expression. B: Western blot analysis of GFAP in the cortex of P8 rats. Top: representative blot images; Bottom: quantitative analysis. C: Western blot analysis of iNOS and CD68 in the cortex of postnatal day 41 (P41) rats. Top: representative blot images; Bottom: quantitative analysis. D, E: Quantification of IL-6 and IL-1β levels by ELISA in the cortex of P8 rats. F, G: Quantification of IL-6 and IL-1β levels by ELISA in the cortex of P41 rats. Data are presented as means ± standard deviation (S.D.), with individual data points representing separate animals (n = 5 per group).

Given the observed microglial activation, we further explored the expression of inflammatory factors in the cortex following repeated sevoflurane exposure. Western blot analysis of P8 rats revealed significantly increased expression of iNOS and CD68 in the SEV group compared with controls (Fig. 3A, iNOS CON [1.06 ± 0.13] vs. SEV [1.61 ± 0.18], *P* = 0.0002; CD68: CON [1.00 ± 0.06] vs. SEV [1.90 ± 0.19], *P* < 0.0001). Baicalein treatment significantly reduced both markers (Fig. 3A, iNOS: SEV [1.61 ± 0.18] vs. SEV + BA [0.95 ± 0.06], *P* < 0.0001; CD68: SEV [1.90 ± 0.19] vs. SEV + BA [1.20 ± 0.19], *P* < 0.0001).

In contrast, no significant differences in iNOS or CD68 expression were detected in P41 rats (Fig. 3C, iNOS: CON [1.00 ± 0.09] vs. SEV [0.95 ± 0.07], *P* = 0.7954; SEV [0.95 ± 0.07] vs. SEV + BA [0.92 ± 0.10], *P* = 0.9359; CD68: CON [1.00 ± 0.11] vs. SEV [0.93 ± 0.07], *P* = 0.6027; SEV [0.93 ± 0.07] vs. SEV + BA [0.87 ± 0.10], *P* = 0.6706).

Immunofluorescence for GFAP revealed a trend toward increased astrocytic activation in P8 rats, although group differences did not reach statistical significance (Fig. 3B, CON [1.00 ± 0.06] vs. SEV [1.27 ± 0.28], *P* = 0.1402; SEV [1.27 ± 0.28] vs. SEV + BA [1.24 ± 0.21], *P* = 0.9912).

ELISA results further confirmed elevated secretion of IL-6 and IL-1β in the cortex of P8 rats after sevoflurane exposure (Fig. 3D and E, IL-6: CON [30.76 ± 9.47] vs. SEV [153.40 ± 44.06], *P* < 0.0001; SEV [153.40 ± 44.06] vs. SEV + BA [35.80 ± 11.56], *P* < 0.0001; IL-1β: CON [63.40 ± 7.25] vs. SEV [210.10 ± 75.92], *P* = 0.0001; SEV [210.10 ± 75.92] vs. SEV + BA [62.80 ± 8.55], *P* = 0.0001). No group differences in cytokine levels were observed in P41 cortices (Fig. 3F and G, IL-6: CON [84.55 ± 14.48] vs. SEV [83.18 ± 12.84], *P* = 0.9986; SEV [83.18 ± 12.84] vs. SEV + BA [105.90 ± 15.42], *P* = 0.0796; IL-1β: CON [89.35 ± 5.77] vs. SEV [81.85 ± 8.00], *P* = 0.4512; SEV [81.85 ± 8.00] vs. SEV + BA [81.63 ± 4.27], *P* > 0.9999).

### 3.3 Baicalein treatment attenuates sevoflurane-induced TLR4/NF-κB activation in cortical microglia of P8 neonatal rats

**Fig. 4:**
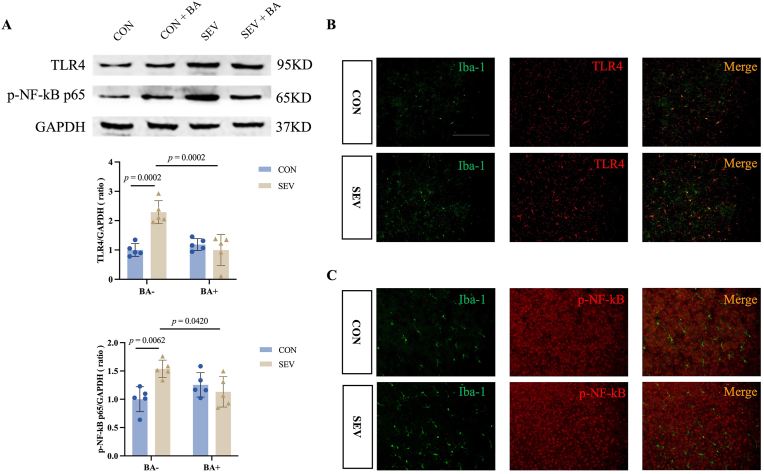
Baicalein treatment attenuates sevoflurane-induced TLR4/NF-κB activation in cortical microglia of P8 neonatal rats. A: Western blotting analysis of TLR4 and phosphorylated NF-κB p65(p-NF-κB p65) in the cortex of P8 rats. Top: Representative images of Western blotting; Bottom: Quantitative analysis of protein expression. Data are presented as means ± standard deviation (S.D.), with individual data points representing separate animals (n = 5 per group). B, C: Representative immunofluorescence images showing co-localization of Iba-1 (microglial marker) with TLR4 and p-NF-κB in the cortex of P8 rats. Scale bar = 100 μm.

To investigate the mechanism underlying microglial activation, we examined the TLR4/NF-κB signaling pathway. Western blot analysis at postnatal day 8 (P8) revealed significant upregulation of TLR4 and p-NF-κB in the SEV group compared with controls (Fig. 4A, TLR4: CON [1.00 ± 0.22] vs. SEV [2.29 ± 0.39], *P* = 0.0002; p-NF-κB p65: CON [1.00 ± 0.22] vs. SEV [1.54 ± 0.15], *P* = 0.0063). Baicalein treatment significantly reduced both TLR4 and p-NF-κB levels (Fig. 4A, TLR4: SEV [2.44 ± 0.44] vs. SEV + BA [1.00 ± 0.53], *P* = 0.0002; p-NF-κB p65: SEV [1.54 ± 0.15] vs. SEV + MEL [1.13 ± 0.27], *P* = 0.0420), indicating that baicalein attenuates early cortical inflammatory signaling induced by neonatal sevoflurane exposure.

Immunofluorescence co-localization further showed that TLR4, but not p-NF-κB, was primarily expressed in Iba-1-positive microglia (Fig. 4B and C). To assess whether TLR4/NF-κB activation persisted into adolescence, we performed immunofluorescence at postnatal day 41 (P41). TLR4 expression was undetectable in the cortex of either control or sevoflurane-exposed rats (data not shown), suggesting that the early neonatal TLR4-mediated inflammatory response resolves by adolescence.

Together, these findings indicate that repeated neonatal sevoflurane exposure transiently activates TLR4/NF-κB signaling in microglia, which is effectively suppressed by baicalein, supporting the notion that baicalein protects against transient microglial overactivation and early cortical inflammation induced by neonatal sevoflurane exposure.

### 3.4 Baicalein rescues sevoflurane-indcuced long-term cognitive impairment following neonatal exposure

**Fig. 5:**
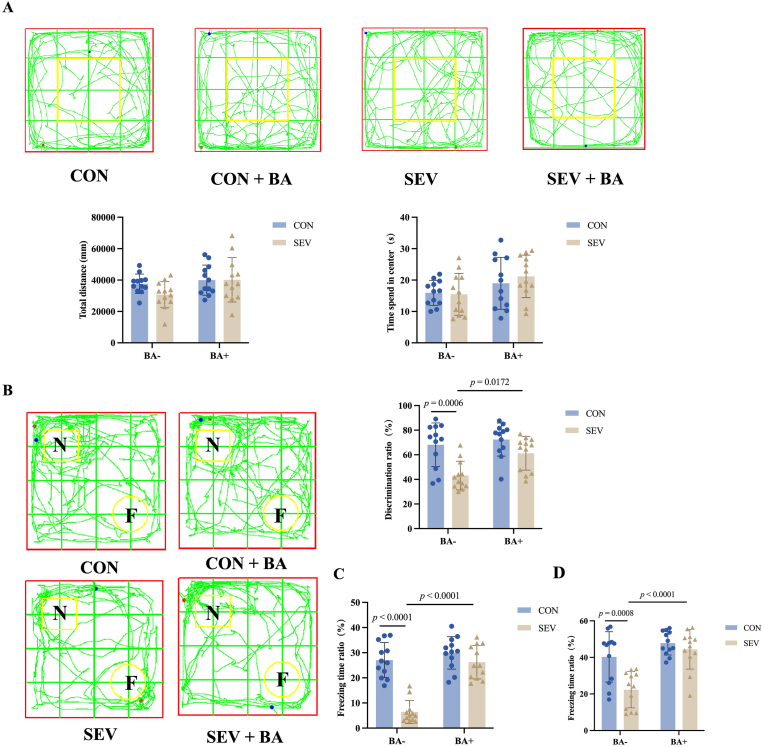
Baicalein rescues sevoflurane indcuced long-term cognitive impairment following neonatal exposure. A: Open field test. Top: representative movement trajectories of rats from each group. Bottom: quantification of total distance traveled and time spent in the center area. B: Novel object recognition (NOR) test. Left: representative exploration trajectories. Right: discrimination index quantifying recognition memory performance. C: Contextual freezing time in the fear conditioning test. D: Cued freezing time in the fear conditioning test. Data are presented as means ± standard deviation (S.D.), with individual data points representing separate animals (n = 12 per group).

To evaluate the long-term effects of baicalein, behavioral tests were conducted in adolescent rats. In the open field test, no significant differences were found in locomotor activity or anxiety-like behavior across groups (Fig. 5A, Total distance: CON [37636 ± 6169] vs. SEV [30735 ± 8404], *P* = 0.3434; SEV [30735 ± 8404] vs. SEV + BA [40198 ± 14186], *P* = 0.1110; Time spend in center: CON [16.46 ± 6.07] vs. SEV [15.48 ± 8.42], *P* = 0.9979; SEV [15.48 ± 8.42] vs. SEV + BA [21.18 ± 8.18], *P* = 0.1633) .

In the novel object recognition test, the SEV group exhibited a significant reduced discrimination index compared with controls (Fig. 5B, CON [68.13 ± 17.77] vs. SEV [43.14 ± 11.66], *P* = 0.0006), which was markedly improved by baicalein (Fig. 5B, SEV [43.14 ± 11.66] vs. SEV + BA [61.25 ± 13.69], *P* = 0.0172).

In the contextual fear conditioning test, freezing time was significantly reduced in the SEV group (Fig. 5C, CON [27.08 ± 6.92] vs. SEV [6.44 ± 4.54], *P* < 0.0001) but was restored by baicalein (Fig. 5C, SEV vs. SEV + BA [26.22 ± 6.64], *P* < 0.0001) Similarly, in the cued fear conditioning test, freezing behavior was impaired in the SEV group but rescued by baicalein (Fig. 5D, CON [40.27 ± 13.76] vs. SEV [22.34 ± 9.95], *P* = 0.0008; SEV vs. SEV + BA [44.44 ± 10.81], *P* < 0.0001).

## 4 Discussion

Neonatal exposure to sevoflurane has been increasingly recognized as a potential risk factor for neurodevelopmental disturbances, particularly during critical periods of brain maturation. Repeated neonatal sevoflurane exposure can trigger transient neuroinflammation that may disrupt normal neural circuit formation and contribute to long-term cognitive deficits (Xia Shen, 2013). Previous studies have reported that baicalein, a flavonoid compound derived from Scutellaria baicalensis, possesses neuroprotective and anti-inflammatory properties, ameliorating cognitive dysfunction in Alzheimer's disease models and modulating sleep and circadian rhythm disturbances (Zisapel, 2018; Howatson et al., 2012; Chen et al., 2021). However, its potential role in mitigating long-term cognitive impairments induced by repeated neonatal sevoflurane exposure has not been systematically investigated.

In line with accumulating evidence supporting baicalein's anti-inflammatory actions (Liu et al., 2024; Gu et al., 2021), our study revealed that repeated neonatal exposure to sevoflurane induced robust cortical inflammation, characterized by increased expression of iNOS, CD68, IL-6, and IL-1β. Baicalein treatment significantly reversed these molecular alterations. Interestingly, GFAP levels remained unchanged, suggesting that microglial activation - rather than astrocytic reactivity - dominates the inflammatory response at this developmental stage. These results are consistent with clinical and preclinical findings that baicalein suppresses pro-inflammatory cytokines and oxidative stress (Cho et al., 2021; El-Missiry et al., 2021). Notably, in adolescent rats (P41), TLR4 immunofluorescence staining yielded negative results, indicating that the acute inflammatory response observed in the neonatal period (P8) had resolved by adolescence. This temporal pattern implies that early-life microglial activation may trigger transient but developmentally significant inflammation that contributes to long-term cognitive consequences.

Microglial activation is a central event linking neuroinflammation to cognitive impairment (Xu et al., 2017). Our data showed that sevoflurane exposure markedly increased Iba-1-positive microglia and pro-inflammatory cytokines in the neonatal cortex, both of which were effectively suppressed by baicalein. These findings suggest that baicalein confers long-term neuroprotection, at least in part, by dampening microglial overactivation and restoring the balance of the neural microenvironment during critical developmental windows.

Mechanistically, we identified the TLR4/NF-κB signaling pathway as a key mediator of the observed microglial activation. Activation of TLR4 triggers downstream NF-κB phosphorylation and nuclear translocation, thereby initiating pro-inflammatory transcriptional programs that contribute to neuronal injury and cognitive deficits (Källstig et al., 2021). In our study, repeated sevoflurane exposures significantly increased TLR4 and phosphorylated NF-κB levels in the neonatal cortex, while baicalein treatment effectively suppressed this pathway. Together with the negative TLR4 immunofluorescence findings at P41, these data suggest that baicalein's neuroprotective effects are primarily exerted during the early inflammatory phase by inhibiting the TLR4/NF-κB cascade, preventing persistent neuroinflammatory signaling and subsequent synaptic dysfunction.

Importantly, our experimental design employed a combined administration strategy. Baicalein was administered via intraperitoneal injection during the sevoflurane exposure period (P6–P8) and via oral administration from P21 to P35. This approach serves two purposes: (1) acute suppression of microglial activation and neuroinflammation during the critical neonatal period through rapid systemic absorption, and (2) sustained intervention during the subsequent developmental window to maintain microglial homeostasis and support synaptic and cognitive recovery. This dual-route strategy ensures maximal neuroprotective efficacy across key stages of brain development and mimics potential clinical strategies involving both acute and maintenance therapy.

Our behavioral findings further support this mechanistic interpretation. Baicalein treatment significantly improved learning and memory performance in adolescent rats subjected to neonatal anesthesia, as evidenced by enhanced performance in open-field, novel object recognition, and fear-conditioning tests. These results align with previous studies showing that baicalein preserves synaptic integrity and mitigates anesthesia-induced cognitive decline in both aged and neonatal models (Liu et al., 2024; Liang et al., 2021).

Collectively, these findings suggest that baicalein exerts its long-term neuroprotective effects by attenuating early-life microglial activation and inflammation through TLR4/NF-κB inhibition, ultimately preserving cognitive function.

## 5 Limitations

This study has several limitations. First, although behavioral tests were conducted in a relatively quiet environment, potential interference from ambient noise could not be entirely excluded. Second, we did not distinguish microglial phenotypes, such as M1 and M2, which warrants further investigation. Third, while microglia are key upstream regulators of inflammation, we did not apply microglia-targeted interventions, which should be addressed in future studies. Fourth, while baicalein was administered both acutely during anesthesia (intraperitoneal) and chronically via oral supplementation, we did not experimentally compare the contributions of these two administration routes. Therefore, it remains unclear whether one route is more effective or whether the observed effects require the combination of both. Finally, only male rats were used to minimize variability; thus, the results may not fully generalize to females, and potential sex-specific effects should be explored in subsequent research.

## 6 Conclusions

**Fig. 6:**
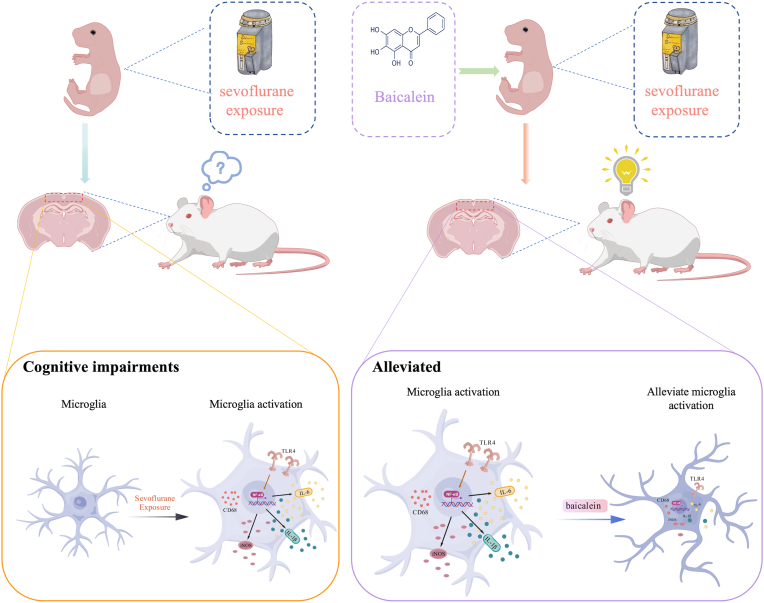
Schematic illustration of the proposed mechanism by which baicalein attenuates long-term cognitive impairments induced by repeated neonatal sevoflurane exposures. Repeated sevoflurane exposures during the neonatal period trigger excessive activation of microglia and promote the release of pro-inflammatory cytokines, including IL-1β, IL-6, and iNOS, leading to neuroinflammation and long-term cognitive deficits. This microglial activation is primarily mediated through the TLR4/NF-κB signaling pathway. Baicalein treatment suppresses microglial activation and inhibits the TLR4/NF-κB pathway, thereby reducing the production of inflammatory cytokines and alleviating cognitive impairments in adolescent rats. The elements in this figure were created using BioGDP.com (https://BioGDP.com).

In conclusion, our study demonstrates that repeated neonatal sevoflurane exposure induces transient microglial activation and TLR4/NF-κB-mediated neuroinflammation, which are associated with long-term cognitive deficits in adolescent rats. Baicalein treatment, administered both acutely during anesthesia and chronically via oral supplementation, effectively suppresses microglial overactivation, reduces pro-inflammatory cytokine release, and restores cognitive performance. These findings highlight the therapeutic potential of baicalein as a multi-stage intervention to prevent anesthesia-related neurodevelopmental impairments, and provide mechanistic insights into microglia- and TLR4/NF-κB-mediated pathways in early-life anesthesia-induced neurotoxicity (see Fig. 6).

## CRediT authorship contribution statement

**Yingqiao Niu:** Writing – original draft, Visualization, Investigation. **Qiuting Zeng:** Visualization, Investigation, Formal analysis. **Yan Yang:** Validation, Methodology. **Wenbo Liu:** Visualization. **Hui Zhang:** Investigation, Conceptualization. **Shuyu Chen:** Writing – review & editing, Validation, Funding acquisition. **Xiaomin Li:** Supervision, Project administration, Methodology, Funding acquisition, Conceptualization.

## Funding

This study was supported by the Youth Program of the National Natural Science Foundation of China (NSFC) (Grant No. 82201425 and 82001153).

## Declaration of competing interest

The authors declare that they have no known competing financial interests or personal relationships that could have appeared to influence the work reported in this paper.

## Data Availability

Data will be made available on request.

## References

[bib1] Ali T., Rahman S.U., Hao Q., Li W., Liu Z., Ali Shah F., Murtaza I., Zhang Z., Yang X., Liu G., Li S. (2020). Melatonin prevents neuroinflammation and relieves depression by attenuating autophagy impairment through FOXO3a regulation. J. Pineal Res..

[bib2] Arioz B.I., Tastan B., Tarakcioglu E., Tufekci K.U., Olcum M., Ersoy N., Bagriyanik A., Genc K., Genc S. (2019). Melatonin Attenuates LPS-induced acute depressive-like behaviors and microglial NLRP3 inflammasome activation through the SIRT1/Nrf2 pathway. Front. Immunol..

[bib3] Chen C., Yang C., Wang J., Huang X., Yu H., Li S., Li S., Zhang Z., Liu J., Yang X., Liu G.P. (2021). Melatonin ameliorates cognitive deficits through improving mitophagy in a mouse model of Alzheimer's disease. J. Pineal Res..

[bib4] Chen B.Z., Jiang L.H., Tan L., Zhou W.Q., Shang Y.C., Li F., Liu B. (2022). Repeated sevoflurane exposures in neonatal rats increased the brain vulnerability to future stress exposure and resulted in fear extinction deficit. Neurotox. Res..

[bib5] Cheng J., He Y., Wang Z., Wang Z., Peng X., Zhang L. (2025). Neonatal sevoflurane exposure exerts sex-specific effects on cognitive function via C3- and TLR4-Related M1/M2 microglial cell polarisation in rats. J. Cell Mol. Med..

[bib6] Cho J.H., Bhutani S., Kim C.H., Irwin M.R. (2021). Anti-inflammatory effects of melatonin: a systematic review and meta-analysis of clinical trials. Brain Behav. Immun..

[bib7] El-Missiry M.A., Shabana S., Ghazala S.J., Othman A.I., Amer M.E. (2021). Melatonin exerts a neuroprotective effect against γ-radiation-induced brain injury in the rat through the modulation of neurotransmitters, inflammatory cytokines, oxidative stress, and apoptosis. Environ. Sci. Pollut. Res. Int..

[bib8] Feldman R.A. (2022). Microglia orchestrate neuroinflammation. eLife.

[bib9] Goyagi T. (2018). The additional oxygen as a carrier gas during long-duration sevoflurane exposure ameliorate the neuronal apoptosis and improve the long-term cognitive function in neonatal rats. Brain Res..

[bib10] Gu C., Wang F., Zhang Y.T., Wei S.Z., Liu J.Y., Sun H.Y., Wang G.H., Liu C.F. (2021). Microglial MT1 activation inhibits LPS-induced neuroinflammation via regulation of metabolic reprogramming. Aging Cell.

[bib11] Howatson G., Bell P.G., Tallent J., Middleton B., McHugh M.P., Ellis J. (2012). Effect of tart cherry juice (Prunus cerasus) on melatonin levels and enhanced sleep quality. Eur. J. Nutr..

[bib12] Jadhav R., Kulkarni Y.A. (2023). The combination of Baicalein and memantine reduces oxidative stress and protects against β-amyloid-Induced Alzheimer's disease in rat model. Antioxidants.

[bib13] Jiang Y., Zhou Y., Tan S., Xu C., Ma J. (2023). Role of posttranslational modifications in memory and cognitive impairments caused by neonatal sevoflurane exposure. Front. Pharmacol..

[bib14] Källstig E., McCabe B.D., Schneider B.L. (2021). The links between ALS and NF-κB. Int. J. Mol. Sci..

[bib15] Lee Jeong-Rim, Joseph Bernadin, Hofacer Rylon D. (2021). Effect of dexmedetomidine on sevoflurane-induced neurodegeneration in neonatal rats. Br. J. Anaesth..

[bib16] Liang L., Zeng T., Zhao Y., Lu R., Guo B., Xie R., Tang W., Zhang L., Mao Z., Yang X., Wu S., Wang Y., Zhang H. (2021). Melatonin pretreatment alleviates the long-term synaptic toxicity and dysmyelination induced by neonatal Sevoflurane exposure via MT1 receptor-mediated Wnt signaling modulation. J. Pineal Res..

[bib17] Liu M.W., Zhang C.H., Ma S.H., Zhang D.Q., Jiang L.Q., Tan Y. (2024). Protective effects of Baicalein on lipopolysaccharide-induced AR42J PACs through attenuation of both inflammation and pyroptosis via downregulation of miR-224-5p/PARP1. Mediat. Inflamm..

[bib18] Liu N., Cui X., Yan W., Guo T., Wang Z., Wei X., Sun Y., Liu J., Xian C., Ma W., Chen L. (2024). Baicalein: a potential GLP-1R agonist improves cognitive disorder of diabetes through mitophagy enhancement. J. Pharm. Anal..

[bib19] Owen Y Chao, Nikolaus Susanne, Yang Yi-Mei, Huston Joseph P. (2022). Neuronal circuitry for recognition memory of object and place in rodent models. Neurosci. Biobehav. Rev..

[bib20] Park B.S., Lee J.O. (2013). Recognition of lipopolysaccharide pattern by TLR4 complexes. Exp. Mol. Med..

[bib21] Poltorak A., He X., Smirnova I., Liu M.Y., Van Huffel C., Du X., Birdwell D., Alejos E., Silva M., Galanos C., Freudenberg M., Ricciardi-Castagnoli P., Layton B., Beutler B. (1998). Defective LPS signaling in C3H/HeJ and C57BL/10ScCr mice: mutations in Tlr4 gene. Science.

[bib22] Qiu L.L., Tan X.X., Yang J.J., Ji M.H., Zhang H., Zhao C., Xia J.Y., Sun J. (2023). Lactate improves long-term cognitive impairment induced by repeated neonatal sevoflurane exposures through SIRT1-mediated regulation of adult hippocampal neurogenesis and synaptic plasticity in Male mice. Mol. Neurobiol..

[bib23] Satomoto M., Sun Z., Adachi Y.U., Makita K. (2018). Neonatal sevoflurane exposure induces adulthood fear-induced learning disability and decreases glutamatergic neurons in the basolateral amygdala. J. Neurosurg. Anesthesiol..

[bib24] Sellgren C.M., Gracias J., Watmuff B., Biag J.D., Thanos J.M., Whittredge P.B., Fu T., Worringer K., Brown H.E., Wang J., Kaykas A., Karmacharya R., Goold C.P., Sheridan S.D., Perlis R.H. (2019). Increased synapse elimination by microglia in schizophrenia patient-derived models of synaptic pruning. Nat. Neurosci..

[bib25] Sen M.K., Mahns D.A., Coorssen J.R., Shortland P.J. (2022). The roles of microglia and astrocytes in phagocytosis and myelination: insights from the cuprizone model of multiple sclerosis. Glia.

[bib26] Shen Xia, Dong Yuanlin, Xu Zhipeng, Wang Hui, Miao Changhong, Soriano Sulpicio G., Sun Dandan, Baxter Mark G., Zhang Yiying, Xie Zhongcong (2013). Selective anesthesia-induced neuroinflammation in developing mouse brain and cognitive impairment. Anesthesiology.

[bib27] Song J., Li M., Kang N., Jin W., Xiao Y., Li Z., Qi Q., Zhang J., Duan Y., Feng X., Lv P. (2024). Baicalein ameliorates cognitive impairment of vascular dementia rats via suppressing neuroinflammation and regulating intestinal microbiota. Brain Res. Bull..

[bib28] Sun Z., Satomoto M., Adachi Y.U., Kinoshita H., Makita K. (2016). Inhibiting NADPH oxidase protects against long-term memory impairment induced by neonatal sevoflurane exposure in mice. Br. J. Anaesth..

[bib29] Tan D.X., Xu B., Zhou X., Reiter R.J. (2018). Pineal calcification, melatonin production, aging, associated health consequences and rejuvenation of the pineal gland. Molecules.

[bib30] Tan X., Wang J., Yao J., Yuan J., Dai Y., Sun M., Zhang T., Yang J., Cai W., Qiu L., Sun J. (2023). Microglia participate in postoperative cognitive dysfunction by mediating the loss of inhibitory synapse through the complement pathway. Neurosci. Lett..

[bib31] Warner D.O., Zaccariello M.J., Katusic S.K., Schroeder D.R., Hanson A.C., Schulte P.J., Buenvenida S.L., Gleich S.J., Wilder R.T., Sprung J., Hu D., Voigt R.G., Paule M.G., Chelonis J.J., Flick R.P. (2018). Neuropsychological and behavioral outcomes after exposure of young children to procedures requiring general Anesthesia: the Mayo Anesthesia safety in kids (MASK) study. Anesthesiology.

[bib32] Won E., Na K.S., Kim Y.K. (2021). Associations between Melatonin, neuroinflammation, and brain alterations in depression. Int. J. Mol. Sci..

[bib33] Xu J., Dong H., Qian Q., Zhang X., Wang Y., Jin W., Qian Y. (2017). Astrocyte-derived CCL2 participates in surgery-induced cognitive dysfunction and neuroinflammation via evoking microglia activation. Behav. Brain Res..

[bib34] Yang Yu, Yu Jiafeng, Wu Banglin (2025). Enriched environment mitigates cognitive impairment in pre-adolescent mice following repeated neonatal sevoflurane exposure by reducing TTBK1 expression and Tau phosphorylation. Neuropharmacology.

[bib35] Yilmaz S., Doğanyiğit Z., Oflamaz A.O., Ateş Ş., Uçar S., Söylemez E.S.A. (2023). Detection of melatonin protective effects in sepsis via argyrophilic nucleolar regulatory region-associated protein synthesis and TLR4/NF-κB signaling pathway. Chem. Biol. Drug Des..

[bib36] Zhang H., Sun X.R., Wang J., Zhang Z.Z., Zhao H.T., Li H.H., Ji M.H., Li K.Y., Yang J.J. (2016). Reactive oxygen species-mediated loss of phenotype of parvalbumin interneurons contributes to long-term cognitive impairments after repeated neonatal ketamine exposures. Neurotox. Res..

[bib37] Zhang H., Sun X., Li J., Shan W., Yang J., Zuo Z. (2023). Endoplasmic Reticulum stress-activated neuronal and microglial autophagy contributes to postoperative cognitive dysfunction in neonatal rats. Neurochem. Res..

[bib38] Zhu B., Liu Y., Hwang S., Archuleta K., Huang H., Campos A., Murad R., Piña-Crespo J., Xu H., Huang T.Y. (2022). Trem2 deletion enhances tau dispersion and pathology through microglia exosomes. Mol. Neurodegener..

[bib39] Zisapel N. (2018). New perspectives on the role of melatonin in human sleep, circadian rhythms and their regulation. Br. J. Pharmacol..

[bib40] Zuo C., Ma J., Pan Y., Zheng D., Chen C., Ruan N., Su Y., Nan H., Lian Q., Lin H. (2022). Isoflurane and sevoflurane induce cognitive impairment in neonatal rats by inhibiting neural stem cell development through microglial activation, neuroinflammation, and suppression of VEGFR2 signaling pathway. Neurotox. Res..

